# Decreased Brain-Derived Neurotrophic Factor, Increased FK506 Binding Protein 5 and Female Sex Are Associated with Multiscale-Assessed Depressive Symptoms During Heart Failure Decompensation: A Polish Prospective Cohort Study

**DOI:** 10.3390/jcm15207893

**Published:** 2026-10-13

**Authors:** Magdalena Zalewska, Karol Nowak, Julia Kościanek, Jadwiga Nessler, Jarosław Zalewski

**Affiliations:** 1 Department of Emergency Medicine, Faculty of Health Sciences, Jagiellonian University Medical College, Michalowskiego 12 Street, 31-126 Krakow, Poland; 2 Department of Thromboembolic Disorders, Institute of Cardiology, Jagiellonian University Medical College, 80 Pradnicka Street, 31-202 Krakow, Poland; 3 Department of Coronary Artery Disease and Heart Failure, Saint John Paul II Hospital, 80 Pradnicka Street, 31-202 Krakow, Poland; 4 Department of Coronary Artery Disease and Heart Failure, Institute of Cardiology, Jagiellonian University Medical College, 80 Pradnicka Street, 31-202 Krakow, Poland

**Keywords:** acute heart failure, depression, brain-derived neurotrophic factor, FK506 binding protein 5, depression screening scales

## Abstract

**Background/Objectives**: Acute decompensated heart failure (ADHF) may be accompanied by depressive symptoms. Risk factors for a screening diagnosis of depression (SDD) in ADHF are poorly understood. **Methods**: We enrolled 51 patients with a median age 67 years hospitalized due to ADHF. SDD was determined by the Beck Depression Inventory (BDI) with >12 points or by the Patient Health Questionnaire-9 (PHQ-9) with >11 points. Severe depressive symptoms were diagnosed based on a Hamilton Depression Rating Scale (HDRS) score of >17 points. Baseline neurotrophic potential and stress response were assessed by plasma brain-derived neurotrophic factor (BDNF) and FK506 binding protein 5 (FKBP5), respectively. **Results**: SDD was identified in 20 (39.2%) patients based on the BDI, in 13 (25.5%) based on the PHQ-9, while severe depressive symptoms according to the HDRS were found in 15 (29.4%) patients. Women had higher BDI (*p* = 0.044), PHQ-9 (*p* = 0.022) and HDRS (*p* = 0.006) scores than men. The BDI score correlated with plasma FKBP5 (r = 0.317, *p* = 0.025) and inversely with BDNF (r = −0.380, *p* = 0.006). In multivariable analysis, a higher BDI score was associated with female sex (β = 0.294, *p* = 0.033) and lower plasma BDNF (β = −0.330, *p* = 0.015), while a higher HDRS score was associated with female sex (β = 0.312, *p* = 0.010) and higher plasma FKBP5 (β = 0.388, *p* = 0.007). **Conclusions**: In a multi-scale assessment, SDD or severe depressive symptoms were identified in 25–40% of ADHF patients. A higher BDI score was independently associated with lower BDNF, whereas a higher HDRS score was associated with female sex and higher FKBP5.

## 1. Introduction

Acute decompensated heart failure (ADHF) is a complex clinical syndrome associated with a significant risk of in-hospital and out-of-hospital complications, disability or death. It remains one of the most common causes of urgent cardiovascular hospitalization [1,2]. Subjects with heart failure (HF), especially those hospitalized due to ADHF, are prone to mental disorders, as highlighted in a recent European Society of Cardiology (ESC) consensus statement [3]. Previous findings indicate that nearly half of patients with chronic heart failure were diagnosed with depression, which was 1.5–2 times more common in women than in men [3,4]. The prevalence of mental disorders, including depressive symptoms, has increased over the last three decades, posing a serious health burden in all countries [5]. Moreover, mental disorders in subjects with HF, most commonly anxiety and depression, may be associated with unfavorable outcomes [3,6].

Recently, several biomarkers have been studied in subjects with HF in order to link the pathophysiological processes leading to cardiac insufficiency with depression [3]. This is even more important in subjects with ADHF, in whom several pathophysiological pathways are intensified, including a prothrombotic state that has been linked to endothelial dysfunction and to the unfavorable long-term prognosis in HF patients with ischemic etiology and reduced systolic function [7,8,9]. A novel molecule linking HF and affective disorders might be brain-derived neurotrophic factor (BDNF), a marker of neuron development and survival that is associated with brain plasticity and has been linked to depression severity measured by validated tests in the general population [10,11,12]. On the other hand, BDNF is also present in other cells, including cardiomyocytes, where it plays a role in cardiac bioenergetics and controls cardiac and vascular growth and development. Accordingly, BDNF has been associated with disease severity and prognosis in patients with chronic HF [13,14,15]. A complementary pathway is represented by the stress-responsive co-chaperone FK506 binding protein 5 (FKBP5), a negative regulator of the glucocorticoid receptor and of hypothalamic–pituitary–adrenal (HPA) feedback, whose dysregulation is a recognized feature of stress-related psychiatric disorders [16,17]. Recently, it was shown that patients with depression responding to antidepressant treatment had a pronounced reduction in FKBP5 gene and FKBP51 protein expression, while increasing expression levels were observed in nonresponders [18].

Diagnosing depression in HF patients remains a significant challenge. Fatigue, sleep disturbance, appetite and weight change, psychomotor slowing, poor concentration, and dyspnea-related anxiety are features of both depression and HF. During an acute episode, these symptoms are amplified by congestion, hypoperfusion, orthopnea, and hospitalization itself. Any single instrument will therefore be confounded to some degree, and the direction of bias depends on how heavily it weights somatic items. Therefore, a multi-scale approach to screening for depressive symptoms in ADHF patients seems particularly promising. The Beck Depression Inventory, first edition (BDI) [19] and the Patient Health Questionnaire-9 (PHQ-9) [20] are self-reported, whereas the Hamilton Depression Rating Scale (HDRS) [21] is administered by a clinician. The BDI places greater emphasis on cognitive–affective symptoms and is less contaminated by HF symptoms, which helps to separate depressed mood from somatic burden. The PHQ-9 is brief, maps directly onto the diagnostic criteria for depression, suits acutely ill patients, and is well validated in cardiac populations. In turn, the clinician-rated HDRS weights more heavily toward somatic, anxiety, and insomnia symptoms and includes suicidality. It contributes an observer’s judgment that does not depend on the patient’s ability or willingness to self-report, which matters when patients are dyspneic, fatigued or cognitively impaired. The HDRS is helpful for rating severity and is therefore useful as a reference or outcome measure. The cut-off points for a screening diagnosis of depression (SDD) have been extensively studied for various scales [22,23]. These three instruments are commonly used in Poland and worldwide, and they differ in the weight they give to somatic symptoms, which overlap considerably with those of decompensated HF and are a recognized reason why depression is both over- and under-detected in cardiac patients [24,25,26].

Although the potential interference between neural and cardiac sources of BDNF alterations and the action of FKBP5 as a negative regulator of the glucocorticoid receptor shaping the brain’s reaction to stress are known, it remains unknown whether low BDNF or high FKBP5 precedes depression, follows hemodynamic severity, or simply marks illness burden [27,28]. Moreover, in patients with decompensated HF, screening for dementia predicted unfavorable long-term outcomes [29]. Therefore, we sought to investigate the independent determinants of depressive symptoms assessed by three validated screening tools in subjects hospitalized with ADHF, including the clinical significance of plasma levels of BDNF and FKBP5.

## 2. Materials and Methods

### 2.1. Study Population

In this prospective study, we enrolled 51 adult patients with the symptoms of ADHF with reduced left ventricular ejection fraction (LVEF) defined as less than 40% according to current ESC guidelines [1], without a prior psychiatric history or antidepressant treatment, who were hospitalized in a tertiary cardiology center between October 2021 and June 2023. The criteria for inclusion were as follows: urgent hospitalization due to symptoms of ADHF, including worsening lower extremity swelling, increasing body weight during the last week, pronounced dyspnea, necessity for oral diuretic dosage increase or administration of intravenous diuretics, an N-terminal prohormone of brain natriuretic peptide (NT-proBNP) level > 500 pg/mL, age over 18 years, sinus rhythm and patient informed consent. The exclusion criteria included chronic anticoagulation due to, e.g., atrial fibrillation, mechanical artificial valves or unprovoked venous thromboembolism; a history of ADHF, myocardial infarction or stroke within the last 3 months; end-stage chronic kidney disease defined as creatinine clearance < 15 mL/min/1.73 m^2^; severe liver dysfunction; diagnosed acute infection; antimicrobial pharmacotherapy; autoimmune disease or immunosuppressive therapy including intake of oral corticosteroids; active neoplastic disease or pregnancy. Diabetes mellitus, chronic kidney disease and hyperlipidemia were diagnosed according to standard criteria. The Bioethics Committee of the Jagiellonian University approved the study protocol (consent no. 1072.6120.350.2020), and all participants provided their written informed consent.

In this prospective study, we enrolled 51 adult patients with the symptoms of ADHF with reduced left ventricular ejection fraction (LVEF) defined as less than 40% according to current ESC guidelines [1], without a prior psychiatric history or antidepressant treatment, who were hospitalized in a tertiary cardiology center between October 2021 and June 2023. The criteria for inclusion were as follows: urgent hospitalization due to symptoms of ADHF, including worsening lower extremity swelling, increasing body weight during the last week, pronounced dyspnea, necessity for oral diuretic dosage increase or administration of intravenous diuretics, an N-terminal prohormone of brain natriuretic peptide (NT-proBNP) level > 500 pg/mL, age over 18 years, sinus rhythm and patient informed consent. The exclusion criteria included chronic anticoagulation due to, e.g., atrial fibrillation, mechanical artificial valves or unprovoked venous thromboembolism; a history of ADHF, myocardial infarction or stroke within the last 3 months; end-stage chronic kidney disease defined as creatinine clearance <15 mL/min/1.73 m^2^; severe liver dysfunction; diagnosed acute infection; antimicrobial pharmacotherapy; autoimmune disease or immunosuppressive therapy including intake of oral corticosteroids; active neoplastic disease or pregnancy. Diabetes mellitus, chronic kidney disease and hyperlipidemia were diagnosed according to standard criteria. The Bioethics Committee of the Jagiellonian University approved the study protocol (consent no. 1072.6120.350.2020), and all participants provided their written informed consent.

### 2.2. Beck Depression Inventory, Patient Health Questionnaire-9 and Hamilton Depression Scale

Depressive symptoms were screened within the first 48 h after hospital admission using validated Polish versions of three instruments, administered by one physician blinded to the biomarker results [30,31,32,33]. In the current study, the most commonly used criteria for SDD were applied [22,23]. The BDI is a 21-item self-report questionnaire. A total score of more than 12 points was considered an SDD [19]. The PHQ-9 comprises nine items rated from 0 to 3 and maps directly onto diagnostic criteria. A cut-off of more than 11 points was applied to identify clinically relevant depressive symptoms [20]. The 17-item HDRS was administered by a trained physician, with scores ≤ 7 points indicating a lack of depression and 8–12, 13–17 and >17 points indicating mild, moderate and severe depression, respectively. Patients with severe symptoms were contrasted with the remaining subjects [21]. In addition, the scores of all three scales were analyzed as continuous variables in relation to other clinical data.

### 2.3. Blood Tests

Blood was drawn between 5 and 8 a.m., after at least 6 h of fasting, from the antecubital vein with minimal stasis within the first 24 h after admission due to acute decompensation of HF. Samples were collected into tubes containing 3.2% trisodium citrate, which were then centrifuged at 2500 *g* for 20 min at 20 °C. Serum containers were spun at 1600 *g* at 4 °C for 10 min. The supernatant was aliquoted and stored at −80 °C. The creatinine, glucose, NT-proBNP, C-reactive protein (CRP) and lipoprotein levels were analyzed on admission by routine hospital techniques.

### 2.4. Brain-Derived Neurotrophic Factor (BDNF), FK506 Binding Protein 5 (FKBP5) and Interleukin 6 (IL-6)

Blood was drawn between 5 and 8 a.m. BDNF, IL-6 and FKBP5 were analyzed according to the manufacturer’s instructions. Plasma samples were first centrifuged at 2–8 °C for 15 min at 1000× *g* within 30 min after collection. BDNF was measured in platelet-poor EDTA plasma. An additional centrifugation step of the separated plasma for complete platelet removal was performed at 10,000× *g* for 10 min at 2–8 °C. Following centrifugation, platelet-poor EDTA plasma was divided into aliquots and stored at −80 °C until analysis. Plasma free BDNF levels were quantified using the Quantikine ELISA kit (R&D Systems, Minneapolis, MN, USA) in duplicate. The intra-assay and inter-assay coefficients of variation were 5.0 and 7.6%, respectively. Total IL-6 levels were quantified in EDTA plasma using the Quantikine ELISA kit (R&D Systems, Minneapolis, MN, USA) in duplicate. The intra-assay and inter-assay coefficients of variation were 2.5 and 4.4%, respectively. FKBP5 was also measured in EDTA plasma and quantified using Human FKBP5 (FK506 Binding Protein 5) ELISA Kit (ELK Biotechnology, Sugar Land, TX, USA) in duplicate. The intra-assay and inter-assay coefficients of variation were 7.1 and 8.0%, respectively. The lower limits of quantification were 0.06 ng/mL for BDNF, 3.13 pg/mL for IL-6 and 0.16 ng/mL for FKBP5.

### 2.5. Echocardiography Examination

The two-dimensional transthoracic echocardiographic examinations were performed in the left decubitus position according to the European Association of Cardiovascular Imaging guidelines. Each echocardiographic examination was performed by a trained physician within the first 48 h of hospitalization using the Epiq 7 ultrasound system (Philips Healthcare, Andover, MA, USA) equipped with a multi-frequency harmonic transducer X5-1 (1–5 MHz).

### 2.6. Statistical Analysis

Statistical analyses were performed using the SPSS Statistics software (Version 31.0.1.0, IBM Corp., Armonk, NY, USA). The study was powered to have an 80% chance of detecting an absolute value of 0.4 for correlation between the BDI score and plasma BDNF in patients with acute HF. To detect a correlation of this magnitude or greater at a significance level of 0.05, 46 patients were required. Continuous variables are presented as mean ± standard deviation or median (interquartile range, IQR), and categorical variables as number (percentage). Normality was assessed using the Shapiro–Wilk test. The in-hospital baseline characteristics of the studied groups were compared by Student’s *t*-test when data were normally distributed or by the Mann–Whitney U test for non-normally distributed parameters. Categorical variables were compared by the Chi-square or Fisher exact test, as appropriate. Pearson or Spearman rank correlation coefficients were calculated to assess the association between two parameters with normal or non-normal distribution, respectively. All independent variables potentially associated with the severity of depressive symptoms at *p* < 0.2 in univariate analysis were entered into multivariable linear regression models to identify independent predictors. A two-tailed *p* < 0.05 was considered statistically significant. For multiple comparisons across the three screening scales, a Bonferroni correction with *p* < 0.016 was applied.

## 3. Results

We enrolled 51 patients (11 women) with a median age of 67 years and LVEF < 40%, hospitalized due to HF decompensation. SDD was identified in 20 (39.2%) patients based on the BDI and in 13 (25.5%) patients based on the PHQ-9. According to the HDRS, 12 (23.5%) patients had no depression, while mild depressive symptoms were found in 18 (35.3%), moderate in 6 (11.8%) and severe in 15 (29.4%) patients.

**Figure 1:**
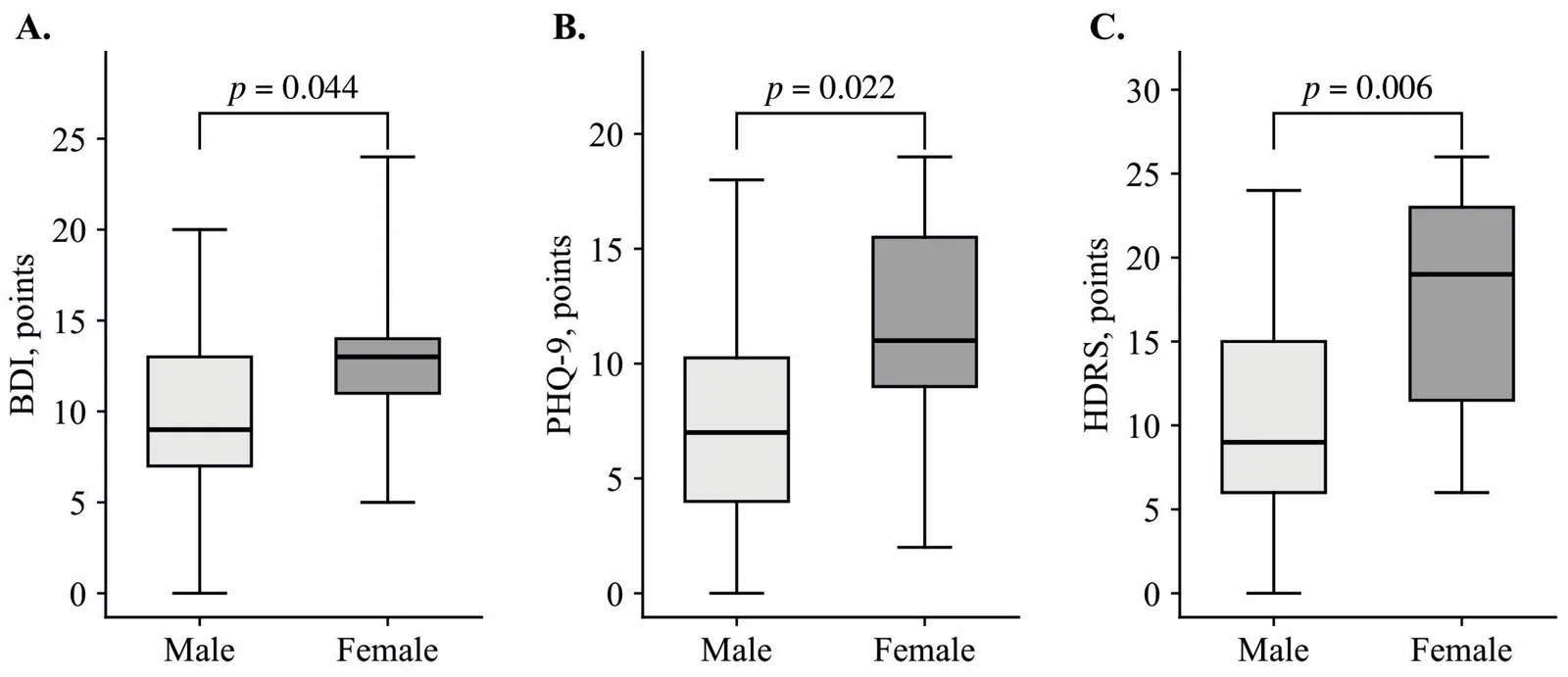
**Scores** of the three depression screening scales according to sex. Abbreviations: (**A**) BDI: Beck Depression Inventory; (**B**) PHQ-9: Patient Health Questionnaire-9; (**C**) HDRS: Hamilton Depression Rating Scale. Box plot shows median and interquartile range (IQR, Q3–Q1). Q1 and Q3 are the first and third quartiles. Whiskers are drawn at minimum and maximum.

Women with HF decompensation had higher BDI (*p* = 0.044), PHQ-9 (*p* = 0.022) and HDRS (*p* = 0.006) scores than men (Figure 1).

**Figure 2:**
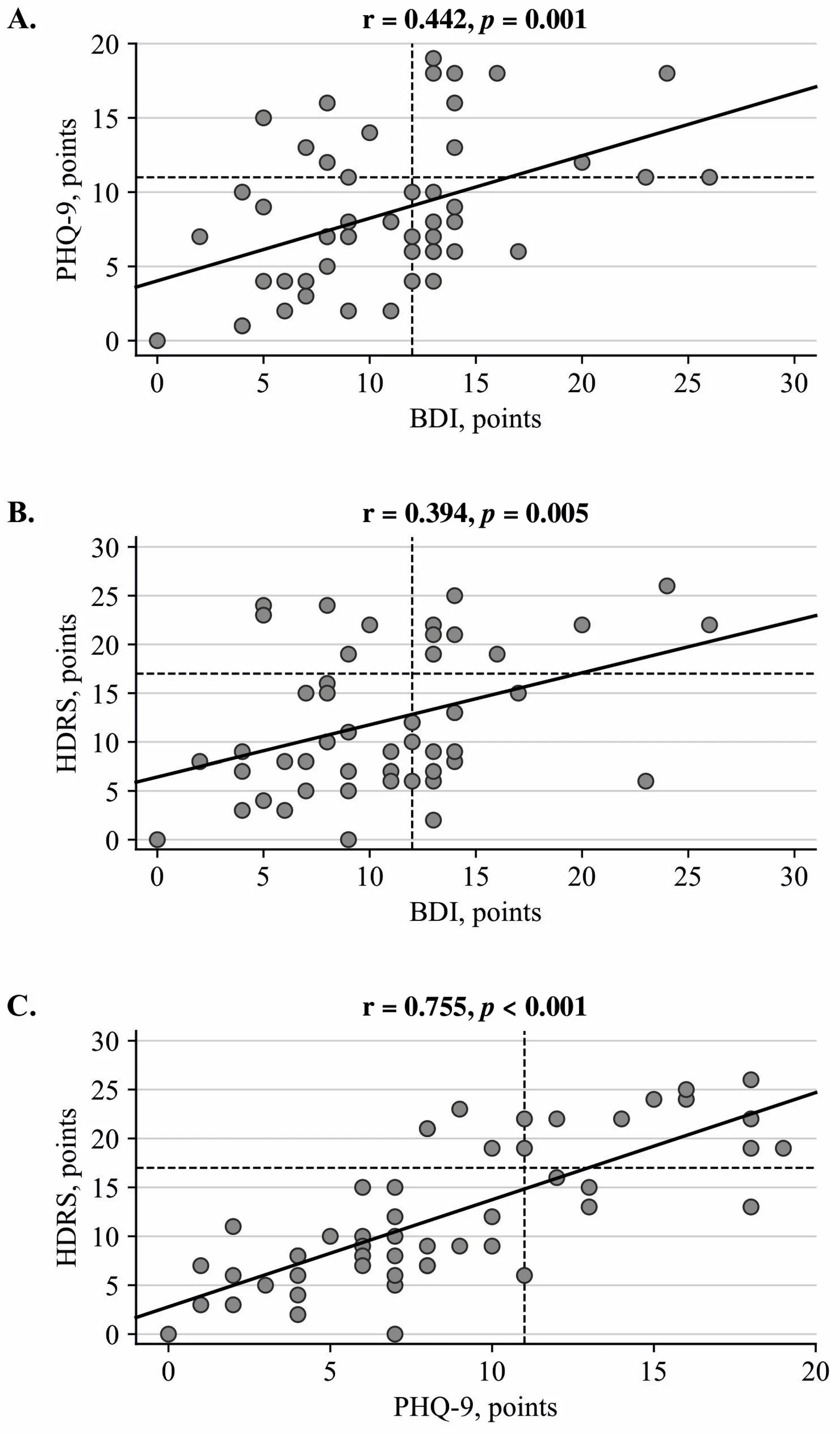
The correlations between the results of three scales used to screen depressive symptoms. Abbreviations: BDI: Beck Depression Inventory, HDRS: Hamilton Depression Rating Scale, PHQ-9: Patient Health Questionnaire-9. In panels (**A**–**C**), the diagonal solid lines indicate regression lines, while the horizontal and vertical dashed lines indicate the cut-off points for the respective scales.

There were moderate correlations between the BDI score and the PHQ-9 (r = 0.442, *p* = 0.001) and the HDRS (r = 0.394, *p* = 0.005), whereas the correlation between the PHQ-9 and HDRS scores was strong (r = 0.755, *p* < 0.001) (Figure 2).

### 3.1. Baseline Characteristics of Patients with SDD Based on Three Screening Scales

The baseline characteristics of patients with and without SDD defined by the BDI are shown in Table 1. Patients with SDD were comparable to those without SDD in terms of age, sex, body mass index and the severity of HF symptoms expressed in New York Heart Association (NYHA) class. The prevalence of cardiovascular risk factors as well as the history of myocardial infarction and stroke were similar in both groups. Laboratory tests revealed a similar distribution of biomarkers of myocardial necrosis, heart failure, renal function and lipid profile. However, patients with SDD had a lower total protein concentration compared with subjects without SDD (*p* = 0.006). Plasma BDNF concentration was also significantly lower in patients with BDI-defined SDD than in those without SDD (*p* = 0.030). The echocardiographic assessment showed no significant differences between groups with and without a BDI-defined SDD.

Grouping the study cohort according to the PHQ-9 criteria for SDD revealed a slightly different profile (Table S1). Patients with a positive PHQ-9 screening result for SDD had higher plasma IL-6 levels (*p* = 0.044), a trend towards higher CRP values (*p* = 0.077) and a lower prevalence of dyslipidemia (69.2 vs. 94.7%, *p* = 0.031), whereas other clinical, laboratory and echocardiography variables were comparable.

When patients with severe depressive symptoms on the HDRS were compared with the remaining subjects (Table 2), the differences were predominantly demographic and structural. Severe symptoms were more frequent in women (*p* = 0.009), were accompanied by a lower prevalence of peripheral arterial disease (*p* = 0.022), a higher triglyceride concentration (*p* = 0.039), a less impaired global longitudinal strain (*p* = 0.024) and a trend towards better LVEF (*p* = 0.088). After adjustment for sex, the difference in global longitudinal strain was no longer significant (*p* = 0.112).

The hospital treatment provided did not differ significantly between groups with and without SDD as assessed by any of the three screening scales.

### 3.2. Univariate Associations with Multi-Scale Defined SDD

In the acute phase, the BDI score correlated with plasma FKBP5 (r = 0.317, *p* = 0.025) and inversely with BDNF (r = −0.380, *p* = 0.006). Neither the PHQ-9 nor the HDRS score correlated with BDNF or FKBP5. In turn, LVEF correlated with the PHQ-9 score (r = 0.329, *p* = 0.018) and with BDNF (r = 0.308, *p* = 0.028) (Table 3). There were no significant associations between BDI, PHQ-9 or HDRS scores and plasma concentrations of NT-proBNP, creatinine, C-reactive protein, interleukin 6 or lipid profile, except for a weak correlation between the HDRS score and triglycerides (r = 0.286, *p* = 0.044). The BDI score was also inversely correlated with total protein (r = −0.324, *p* = 0.025) and albumin (r = −0.286, *p* = 0.042).

The median volume of fluid removed was 3.3 L (interquartile range 2.1–8.4 L) and correlated with admission NT-proBNP (r = 0.38, *p* = 0.006). Neither decongestion volume nor admission hemoglobin or hematocrit was associated with plasma BDNF, total protein or the BDI score (for all *p* > 0.25). In contrast, albumin was inversely correlated with decongestion volume (r = −0.42, *p* = 0.002). After adjustment for decongestion volume and admission hemoglobin, the association between BDNF and the BDI score remained significant (r = −0.326, *p* = 0.043). The interaction between BDNF and decongestion volume was not significant (*p* = 0.834).

### 3.3. Independent Predictors of Multi-Scale Defined SDD

Before inclusion in the multivariable models, correlations between candidate independent variables were assessed. There were univariate correlations between total protein and albumin (r = 0.614, *p* < 0.001), total protein and C-reactive protein (r = −0.342, *p* = 0.033), total protein and body mass index (r = 0.354, *p* = 0.032), left ventricular ejection fraction and global longitudinal strain (r = −0.646, *p* < 0.001) and left ventricular ejection fraction and end-diastolic volume (r = −0.358, *p* = 0.022). Considering these collinearities, the models explaining the highest proportion of variance in the dependent variable were selected. By multivariable linear regression, the higher result of the BDI was associated with female sex (β = 0.294, *p* = 0.033), with a lower plasma BDNF level (β = −0.330, *p* = 0.015), and with a lower total plasma protein concentration (β = −0.260, *p* = 0.049) (Table 4A). The higher result of the HDRS was associated with female sex (β = 0.312, *p* = 0.010), higher left ventricular ejection fraction (β = 0.294, *p* = 0.033) and a higher plasma level of FKBP5 (β = 0.388, *p* = 0.007) (Table 4B). In turn, the PHQ-9 score was independently associated with female sex (β = 0.257, *p* = 0.048), higher left ventricular ejection fraction (β = 0.407, *p* = 0.005), a higher plasma level of IL-6 (β = 0.361, *p* = 0.009) and lower triglyceride levels (β = −0.291, *p* = 0.032) (Table 4C).

## 4. Discussion

This study demonstrated that, depending on the depression screening tool used, between one-quarter and two-fifths of hospitalized patients with symptoms of acute decompensated heart failure had an SDD or severe depressive symptoms. Regardless of the scale used, more severe depressive symptoms were observed in women, even though they constituted only one-fifth of the study group. Moreover, lower plasma BDNF and higher FKBP5 levels were independently associated with more pronounced depressive symptoms on the BDI and HDRS, respectively. An additional observation, which was not anticipated at the stage of study design, is that the three screening instruments applied to the same cohort did not behave interchangeably; each was linked to a separate set of clinical and biochemical variables. Our results address a knowledge gap regarding BDNF and FKBP5 in ADHF and their association with depressive symptoms assessed by multiple screening tools.

Depressive symptoms were more pronounced in women on all three instruments, and female sex remained independently associated with depressive symptom severity. This is in line with population data showing an approximately two-fold higher prevalence of depression in women, and with the ESC consensus statement, which emphasizes that the interaction between mental health and cardiovascular diseases is modified by sex [3]. The observation is nonetheless worth underlining in the context of HF with reduced LVEF, a male-dominated population in which female patients are consequently under-represented in trials and may be screened less systematically. The fact that the difference was evident on the clinician-rated HDRS as well as on the self-reported questionnaires and in previous studies [34] argues against it being merely a reporting artifact, although the small number of women limits the precision of the effect size and warrants confirmation in larger cohorts.

The divergence between the scales is best explained by what each instrument measures and how [22,23,25,26]. In our cohort, the three instruments captured partly non-overlapping correlates within the same population including nutritional for the BDI, inflammatory for the PHQ-9, and demographic and structural for the HDRS. The somatic and cognitive dimensions of depression carry different prognostic weight in HF. The somatic dimension, dominated by fatigue and loss of appetite, predicts mortality in chronic heart failure, but the cognitive dimension does not [35,36]. Moreover, even a two-item PHQ version administered during hospitalization identified patients at increased risk of 12-month mortality [37]. A systematic review of depression screening in cardiovascular care concluded that the final effect depends mainly on the instrument and threshold chosen and that screening improves outcomes only when coupled to management [38]. Our multivariable regression results indicate that, regardless of the screening tool used, female sex was associated with the severity of depressive symptoms. Furthermore, a higher BDI score was associated with lower plasma BDNF levels and lower plasma total protein levels. In turn, a higher HDRS score was associated with higher LVEF and higher plasma FKBP5 levels, whereas the PHQ-9 score was associated with higher LVEF, higher IL-6 levels, and lower triglyceride levels. These discrepancies likely reflect differences in the psychometric properties of the three scales applied. This argues against relying on a single questionnaire in ADHF and supports pairing a self-report with a clinician assessment whenever depressive symptoms are suspected.

The inverse association between BDNF and the result of the BDI is consistent with the reduced serum BDNF reported in untreated patients with major depression and with its normalization after effective treatment [10,12,39,40]. Interpreting circulating BDNF in HF is further complicated by its dual origin. Beyond the neuronal source, BDNF is expressed in cardiomyocytes and vascular cells, where it supports cardiac bioenergetics and vascular homeostasis [41]. Low serum BDNF has been associated with reduced exercise capacity and with an increased risk of death and rehospitalization in HF [13,14,15]. The trend towards positive correlation between left ventricular function and BDNF observed in this study is more likely an indicator of a partially cardiac origin of BDNF than a causal effect of contractile function on mood. This finding cautions against attributing peripheral BDNF solely to central nervous system activity. However, the inverse association of BDNF with the BDI score may suggest a component that is not explained by systolic cardiac function. Moreover, after adjustment for decongestion volume and admission hemoglobin, the association between BDNF and the BDI score remained significant. A screening diagnosis of dementia after HF decompensation has itself been associated with concentric left ventricular remodeling and a higher relative cardiac wall thickness [42]. Finally, depression as a phenomenon accompanying severe cardiac dysfunction and depression as an independent disorder may require different therapeutic approaches [43,44].

The parallel association of depressive symptoms with FKBP5 points to the HPA axis as a complementary mechanism. FKBP5 encodes FKBP51, a co-chaperone in the Hsp90 complex that holds the glucocorticoid receptor in a low-affinity state and limits its nuclear translocation. FKBP5 transcription induced through glucocorticoid response elements forms an ultra-short intracellular negative-feedback loop that dampens glucocorticoid receptor signaling [16]. When that loop is set too high, it creates relative glucocorticoid receptor resistance, sustained HPA axis activation, and delayed recovery from stress. This mechanism is plausibly shared across post-traumatic stress disorder, major depression, anxiety disorders, and some psychotic and substance use phenotypes [17,18]. In turn, sustained glucocorticoid signaling suppresses hippocampal BDNF transcription, providing a mechanistic link between BDNF and FKBP5, which behaved in opposite directions in our cohort. Moreover, glucocorticoid resistance sustains a pro-inflammatory state that is itself associated with depressive symptoms [45]. Elevated IL-6, observed in our study in subjects with PHQ-9-defined SDD, is one of the most reproducible immunological findings in major depression [46], and its appearance with the somatic PHQ-9, rather than with the Beck inventory, suggests that inflammation is in this case more closely related to the somatic than the cognitive–affective expression of low mood.

Regardless of the association between circulating BDNF and FKBP5 and depressive symptoms, given the cross-sectional nature of this study, it should be emphasized that BDNF and FKBP5 cannot be interpreted as causal mediators of depression, but rather as components of a complex biological network underlying both acute HF and mood symptoms. The ESC recognizes mental disorders as major determinants of cardiovascular risk and encourages collaboration with the European Psychiatric Association [47]. Therefore, systematic multi-scale screening of depressive symptoms may facilitate multidisciplinary management, improve treatment adherence, optimize rehabilitation, and ultimately influence cardiovascular outcomes [3]. Moreover, consistent with previous findings, our results identified female sex as an independent determinant of depressive symptom severity across various screening tools, suggesting that sex-specific biological and psychosocial mechanisms may contribute to depressive symptoms in ADHF [4].

An additional and, to our knowledge, rarely reported observation is the association of the Beck-defined SDD with lower albumin and total protein. Both are markers of the catabolic state that accompanies advanced HF and cardiac cachexia, and both are driven in part by the same inflammatory signaling that suppresses appetite. Anorexia and weight loss are simultaneously items of the depression inventories and features of decompensation, so this association may represent shared biology of both disorders. Moreover, serum BDNF levels were significantly reduced in underweight women with anorexia nervosa and in normal-weight women with bulimia nervosa, but not in overweight women with binge-eating disorder [48]. In turn, in elderly patients with cancer receiving first-line chemotherapy, baseline depressive symptoms and malnutrition or risk of malnutrition were independent predictors of subsequent depressive symptoms [49]. Therefore, nutritional status deserves attention when depressive symptoms are identified during HF decompensation, especially since hypoalbuminemia itself is associated with adverse outcomes in HF.

Our findings provide additional arguments for the need for structured screening for depressive symptoms during HF decompensation, which carries prognostic relevance [27], demonstrating also that the choice of tool influences which patients are identified [3]. In turn, randomized trials of selective serotonin-reuptake inhibitors in HF—SADHART-CHF with sertraline [50] and MOOD-HF with escitalopram [51]—improved neither depressive symptoms nor cardiovascular outcomes beyond usual care, and attention has accordingly shifted towards mechanism-based and behavioral strategies. Exercise training is of particular interest here, because it produces a modest but significant reduction in depressive symptoms in chronic HF [52] and is one of the few interventions known to raise BDNF expression [53]. Whether the neurotrophic and stress-response markers studied here can identify patients in whom depression is biologically anchored, and who might therefore benefit from such an approach, is a question for prospective work. Since BDNF and FKBP5 have a number of mechanistic targets in both the nervous and the cardiovascular system, they may be particularly useful at the interface between these two systems. This issue requires further, extensive research.

This study has several limitations. First, the sample was small and drawn from a single tertiary center, which limits statistical power and generalizability. Second, depression was defined by validated screening instruments rather than by a structured psychiatric interview, so a screening diagnosis does not equate to clinical major depression. Third, the analysis was cross-sectional and cannot establish the direction of the observed associations. Fourth, the absence of a plasma cortisol measurement precludes interpretation of the FKBP5 result in the context of HPA axis function. Although numerous studies have assessed FKBP5 mRNA or protein in blood cells, plasma FKBP5 has not been clinically validated. Fifth, no age- and sex-matched control group without HF was analyzed to compare the BDNF and FKBP5 concentrations. Finally, the number of comparisons performed raises the possibility of type I error, so the weaker associations should be treated as exploratory. Therefore, the study findings require external validation.

In conclusion, in a multi-scale assessment, between one-quarter and two-fifths of patients hospitalized with symptoms of acute HF decompensation had an SDD or severe depressive symptoms. Regardless of the scale applied, and despite the limited sample size, the severity of depressive symptoms was associated with female sex. Moreover, more severe depressive symptoms were associated with lower plasma BDNF and higher FKBP5 on the BDI and HDRS, respectively. The clinical correlates of depressive symptoms differed between the screening scales, underlining that the instrument used affects which patients are identified and supporting a multi-scale approach to depression in acute HF.

## Figures and Tables

**Table 1:** Baseline characteristics of patients with and without a screening diagnosis of depression based on the Beck Depression Inventory.

	BDI-Defined SDD (*n* = 20)	BDI-Defined No SDD (*n* = 31)	*p*-Value
**Clinical characteristics**			
Age, years	69.5 (52.0–73.8)	69.0 (61.0–74.0)	0.802
Female sex	6 (30.0)	5 (16.1)	0.304
Body mass index, kg/m^2^	26.2 (23.6–30.2)	28.7 (26.1–31.3)	0.199
NYHA III/IV	16 (80.0)	24 (77.4)	1.000
Ischemic etiology	9 (45.0)	18 (58.1)	0.532
Prior myocardial infarction	7 (35.0)	15 (48.4)	0.514
Prior stroke	0 (0.0)	3 (9.7)	0.271
Arterial hypertension	16 (80.0)	28 (90.3)	0.411
Dyslipidemia	19 (95.0)	26 (83.9)	0.384
Diabetes mellitus	11 (55.0)	15 (48.4)	0.862
Renal failure	8 (40.0)	6 (19.4)	0.196
Peripheral arterial disease	3 (15.0)	8 (25.8)	0.493
**Laboratory**			
NT-proBNP, pg/mL	7128 (2343–13,041)	4857 (1970–7568)	0.340
Hemoglobin, g/dL	14.1 (12.3–15.3)	13.8 (12.8–14.6)	0.714
Creatinine, µmol/L	118.4 ± 45.6	105.4 ± 34.7	0.286
C-reactive protein, mg/L	10.7 (4.1–18.8)	7.9 (2.5–12.0)	0.568
Total cholesterol, mmol/L	3.8 ± 1.3	4.2 ± 1.1	0.301
LDL cholesterol, mmol/L	2.4 ± 1.1	2.4 ± 0.9	0.962
Triglycerides, mmol/L	1.2 (1.1–1.5)	1.1 (0.9–1.4)	0.384
Albumin, g/L	36.4 ± 4.7	38.7 ± 4.4	0.085
Total protein, g/L	60.3 ± 7.4	67.1 ± 8.4	**0.006**
Interleukin 6, pg/mL	78.9 (0.0–224.3)	102.1 (26.1–149.2)	0.808
BDNF, ng/mL	4.21 ± 1.09	4.95 ± 1.25	**0.030**
FKBP5, ng/mL	0.4 (0.1–0.9)	0.2 (0.1–0.5)	0.111
**Echocardiography**			
LV ejection fraction, %	30.5 (20.0–37.2)	25.0 (20.0–30.0)	0.214
LV end-diastolic diameter, mm	63.1 ± 7.0	60.7 ± 7.9	0.257
Interventricular septum, mm	10.0 (8.8–11.2)	10.0 (10.0–11.0)	0.526
LV mass index, g/m^2^	127.8 (103.0–165.5)	128.6 (105.8–142.8)	0.707
LV end-diastolic volume, mL	220.2 ± 55.7	196.4 ± 35.5	0.197
Global longitudinal strain, %	−8.1 ± 3.5	−7.6 ± 3.6	0.690
Left atrium diameter, mm	47.0 ± 6.4	46.0 ± 7.0	0.632
TAPSE, mm	18.9 ± 5.3	18.5 ± 5.3	0.792
RVSP, mmHg	40.5 (21.8–47.2)	33.0 (18.0–45.5)	0.589

Abbreviations: data are shown as mean ± SD, median (interquartile range) or number (percentage). BDI: Beck Depression Inventory, SDD: screening diagnosis of depression based on the BDI with more than 12 points, NYHA: New York Heart Association, NT-proBNP: N-terminal prohormone of brain natriuretic peptide, LDL: low-density lipoprotein cholesterol, IL-6: interleukin 6, BDNF: brain-derived neurotrophic factor, FKBP5: FK506 binding protein 5; LV: left ventricular; TAPSE: tricuspid annular plane systolic excursion; RVSP: right ventricular systolic pressure.

**Table 2:** Characteristics of patients with severe versus non-severe depressive symptoms based on the Hamilton Depression Rating Scale.

	Severe Depressive Symptoms (*n* = 15)	Non-Severe Depressive Symptoms (*n* = 36)	*p*-Value
**Clinical characteristics**			
Age, years	68.0 (47.0–75.5)	69.5 (61.0–74.0)	0.598
Female sex	7 (46.7)	4 (11.1)	**0.009**
Body mass index, kg/m^2^	25.6 (23.8–29.0)	28.7 (26.6–30.8)	0.119
NYHA III/IV	11 (78.6)	29 (93.5)	0.711
Ischemic etiology	6 (40.0)	21 (58.3)	0.375
Prior myocardial infarction	5 (33.3)	17 (47.2)	0.547
Prior stroke	0 (0.0)	3 (8.3)	0.546
Arterial hypertension	12 (80.0)	32 (88.9)	0.406
Dyslipidemia	11 (73.3)	34 (94.4)	0.054
Diabetes mellitus	7 (46.7)	19 (52.8)	0.928
Renal failure	3 (20.0)	11 (30.6)	0.513
Peripheral arterial disease	0 (0.0)	11 (30.6)	**0.022**
**Laboratory**			
NT-proBNP, pg/mL	3422 (1686–14,155)	5594 (2415–9870)	0.877
Hemoglobin, g/dL	14.0 ± 2.0	13.4 ± 1.8	0.362
Creatinine, µmol/L	97.0 (78.5–111.5)	101.0 (83.0–139.0)	0.415
C-reactive protein, mg/L	4.9 (3.2–14.7)	9.4 (3.0–17.2)	0.791
Total cholesterol, mmol/L	4.1 ± 1.1	4.0 ± 1.3	0.895
LDL cholesterol, mmol/L	2.2 ± 0.8	2.5 ± 1.0	0.307
Triglycerides, mmol/L	1.3 (1.1–3.0)	1.1 (0.9–1.3)	**0.039**
Albumin, g/L	37.5 ± 6.3	37.9 ± 3.8	0.794
Total protein, g/L	61.6 ± 10.5	65.5 ± 7.6	0.224
Interleukin 6, pg/mL	128.5 (75.7–210.1)	68.1 (0.0–151.7)	0.097
BDNF, ng/mL	4.56 ± 1.27	4.71 ± 1.23	0.707
FKBP5, ng/mL	0.2 (0.1–0.7)	0.3 (0.1–0.6)	0.893
**Echocardiography**			
LV ejection fraction, %	30.0 (25.0–36.5)	24.0 (19.5–31.5)	0.088
LV end-diastolic diameter, mm	61.9 ± 6.8	61.5 ± 8.0	0.879
Interventricular septum, mm	10.0 (9.0–10.5)	11.0 (10.0–12.0)	0.117
LV mass index, g/m^2^	119.7 (105.1–142.8)	128.8 (104.6–147.2)	0.877
LV end-diastolic volume, mL	206.4 ± 52.9	204.1 ± 42.4	0.913
Global longitudinal strain, %	−9.8 ± 2.7	−6.9 ± 3.5	**0.024**
Left atrium diameter, mm	47.0 (42.5–51.5)	44.5 (42.0–50.2)	0.460
TAPSE, mm	20.4 ± 3.4	18.1 ± 5.7	0.102
RVSP, mmHg	35.0 (21.0–47.5)	35.5 (18.0–46.2)	0.687

Abbreviations: data are shown as mean ± SD, median (interquartile range) or number (percentage). Severe depressive symptoms based on the Hamilton Depression Rating Scale with more than 17 points. NYHA: New York Heart Association; NT-proBNP: N-terminal prohormone of brain natriuretic peptide; LDL: low-density lipoprotein cholesterol; BDNF: brain-derived neurotrophic factor; FKBP5: FK506 binding protein 5; LV: left ventricular; TAPSE: tricuspid annular plane systolic excursion; RVSP: right ventricular systolic pressure.

**Table 3:** The correlations between the results of the three scales used for depressive symptoms screening and blood tests.

		BDI	HDRS	PHQ-9
Age	r	0.049	0.015	0.046
	*p*-value	0.734	0.916	0.747
Body mass index	r	−0.062	−0.214	−0.051
	*p*-value	0.668	0.132	0.721
NT-proBNP	r	0.098	−0.018	0.003
	*p*-value	0.495	0.899	0.986
Creatinine	r	0.079	−0.196	−0.127
	*p*-value	0.584	0.173	0.379
C-reactive protein	r	0.258	0.118	0.160
	*p*-value	0.113	0.475	0.330
Interleukin 6	r	0.045	0.235	0.149
	*p*-value	0.755	0.097	0.296
Total cholesterol	r	−0.187	−0.001	0.013
	*p*-value	0.195	0.995	0.930
LDL cholesterol	r	−0.145	−0.126	−0.203
	*p*-value	0.316	0.385	0.158
Triglycerides	r	0.046	**0.286**	0.207
	*p*-value	0.752	**0.044**	0.149
Total protein	r	**−0.324**	−0.112	−0.169
	*p*-value	**0.025**	0.447	0.251
Albumin	r	**−0.286**	0.007	−0.142
	*p*-value	**0.042**	0.964	0.321
BDNF	r	**−0.380**	−0.039	0.007
	*p*-value	**0.006**	0.788	0.963
FKBP5	r	**0.317**	0.181	0.138
	*p*-value	**0.025**	0.202	0.334
LVEF	r	0.144	0.198	**0.329**
	*p*-value	0.314	0.164	**0.018**

Abbreviations: BDI: Beck Depression Inventory, HDRS: Hamilton Depression Rating Scale, PHQ-9: Patient Health Questionnaire-9, NT-proBNP: N-terminal prohormone of brain natriuretic peptide, LDL: low-density lipoprotein cholesterol, BDNF: brain-derived neurotrophic factor; FKBP5: FK506 binding protein 5, LVEF: left ventricular ejection fraction.

**Table 4:** The independent predictors of the result of the Beck Depression Inventory (**A**), the Hamilton Depression Rating Scale (**B**) and the Patient Health Questionnaire-9 (**C**) as dependent variables.

**A**	**Univariate Regression**				**Multivariable Regression**			
R^2^ = 0.30, *p* = 0.001	Beta coefficient	Lower 95% CI	Upper 95% CI	*p*-value	Beta coefficient	Lower 95% CI	Upper 95% CI	*p*-value
Sex, female vs. male	0.273	−0.068	0.549	0.052	0.294	0.032	0.551	**0.033**
BDNF, per 1 ng/mL	−0.345	−0.598	−0.067	**0.013**	−0.330	−0.581	−0.083	**0.015**
Total protein, per 1 g/L	−0.249	−0.540	0.038	0.088	−0.260	−0.513	−0.007	**0.049**
Total cholesterol, per 1 mmol/L	−0.222	−0.713	0.316	0.121	−0.202	−0.751	0.354	0.137
**B**	**Univariate regression**				**Multivariable regression**			
R^2^ = 0.31, *p* = 0.002	Beta coefficient	Lower 95% CI	Upper 95% CI	*p*-value	Beta coefficient	Lower 95% CI	Upper 95% CI	*p*-value
Sex, female vs. male	0.403	0.140	0.667	**0.003**	0.312	0.100	0.552	**0.010**
FKBP5, per 1 ng/mL	0.251	−0.027	0.529	0.076	0.388	0.071	0.701	**0.007**
LVEF, per 1%	0.210	−0.072	0.492	0.140	0.294	0.052	0.564	**0.033**
Triglycerides, per 1 mmol/L	0.196	−0.050	0.391	0.085	0.137	−0.070	0.350	0.234
**C**	**Univariate regression**				**Multivariable regression**			
R^2^ = 0.33, *p* = 0.002	Beta coefficient	Lower 95% CI	Upper 95% CI	*p*-value	Beta coefficient	Lower 95% CI	Upper 95% CI	*p*-value
Sex, female vs. male	0.332	0.088	0.451	**0.017**	0.257	0.040	0.478	**0.048**
Interleukin 6, per 1 pg/mL	0.197	−0.015	0.543	0.165	0.361	0.053	0.671	**0.009**
LVEF, per 1%	0.292	0.021	0.585	**0.038**	0.407	0.089	0.725	**0.005**
Triglycerides, per 1 mmol/L	−0.218	−0.598	0.170	0.162	−0.291	−0.530	−0.060	**0.032**

Abbreviations: CI: confidence interval; LVEF: left ventricular ejection fraction; BDNF: brain-derived neurotrophic factor; FKBP5: FK506 binding protein 5.

## Data Availability

The data presented in this study are available on reasonable request from the corresponding author.

## Supplementary Materials

The following supporting information can be downloaded at: https://www.mdpi.com/article/10.3390/jcm15207893/s1, Table S1: Characteristics of patients with (>11 points) and without (≤11 points) a positive PHQ-9 screening.

## References

[1] McDonagh T.A., Metra M., Adamo M., Gardner R.S., Baumbach A., Böhm M., Burri H., Butler J., Čelutkienė J., Chioncel O. (2021). ESC Scientific Document Group. 2021 ESC Guidelines for the diagnosis and treatment of acute and chronic heart failure. Eur. Heart J..

[2] Vaccarino V., Prescott E., Shah A.J., Bremner J.D., Raggi P., Dobiliene O., Gale C.P., Bugiardini R. (2025). Mental health disorders and their impact on cardiovascular health disparities. Lancet Reg. Health Eur..

[3] Bueno H., Deaton C., Farrero M., Forsyth F., Braunschweig F., Buccheri S., Dragan S., Gevaert S., Held C., Kurpas D. (2025). 2025 ESC Clinical Consensus Statement on mental health and cardiovascular disease: Developed under the auspices of the ESC Clinical Practice Guidelines Committee. Eur. Heart J..

[4] Gottlieb S.S., Khatta M., Friedmann E., Einbinder L., Katzen S., Baker B., Marshall J., Minshall S., Robinson S., Fisher M.L. (2004). The influence of age, gender, and race on the prevalence of depression in heart failure patients. J. Am. Coll. Cardiol..

[5] GBD 2023 Mental Disorder Collaborators (2026). Updated trends in the global prevalence and burden of mental disorders, 1990-2023: A systematic analysis for the Global Burden of Disease Study 2023. Lancet.

[6] Rutledge T., Reis V.A., Linke S.E., Greenberg B.H., Mills P.J. (2006). Depression in heart failure: A meta-analytic review of prevalence, intervention effects, and associations with clinical outcomes. J. Am. Coll. Cardiol..

[7] Nowak K., Siniarski A., Karcińska A., Yika A.D.C., Matrejek A., Stępień K., Nessler J., Gajos G., Zalewski J. (2026). Prothrombotic potential in patients with ischemic heart failure: An association with endothelial dysfunction. Adv. Med. Sci..

[8] Nowak K.W., Żmudzki P., Karcińska A., Yika A.D.C., Matrejek A., Stępień K., Furman-Niedziejko A., Nessler J., Zalewski J. (2026). Altered fibrin clot properties and long-term mortality are associated with biventricular decompensation in heart failure with reduced contractility. Thromb. Haemost..

[9] Nowak K., Ząbczyk M., Natorska J., Polak M., Zalewski J., Undas A. (2025). Altered fibrin clot properties and elevated von Willebrand factor are associated with progression to permanent atrial fibrillation: A cohort study. Eur. J. Clin. Investig..

[10] Molendijk M.L., Spinhoven P., Polak M., Bus B.A., Penninx B.W., Elzinga B.M. (2014). Serum BDNF concentrations as peripheral manifestations of depression: Evidence from a systematic review and meta-analyses on 179 associations (N = 9484). Mol. Psychiatry.

[11] Duman R.S., Monteggia L.M. (2006). A neurotrophic model for stress-related mood disorders. Biol. Psychiatry.

[12] Sen S., Duman R., Sanacora G. (2008). Serum brain-derived neurotrophic factor, depression, and antidepressant medications: Meta-analyses and implications. Biol. Psychiatry.

[13] Shibata A., Hanatani A., Izumi Y., Kitada R., Iwata S., Yoshiyama M. (2018). Serum brain-derived neurotrophic factor level and exercise tolerance complement each other in predicting the prognosis of patients with heart failure. Heart Vessel..

[14] Barman H.A., Şahin I., Atıcı A., Durmaz E., Yurtseven E., Ikitimur B., Okuyan E., Keleş I. (2019). Prognostic significance of brain-derived neurotrophic factor levels in patients with heart failure and reduced left ventricular ejection fraction. Anatol. J. Cardiol..

[15] Kadowaki S., Shishido T., Honda Y., Narumi T., Otaki Y., Kinoshita D., Nishiyama S., Takahashi H., Arimoto T., Miyamoto T. (2016). Additive clinical value of serum brain-derived neurotrophic factor for prediction of chronic heart failure outcome. Heart Vessel..

[16] Binder E.B. (2009). The role of FKBP5, a co-chaperone of the glucocorticoid receptor in the pathogenesis and therapy of affective and anxiety disorders. Psychoneuroendocrinology.

[17] Feinmesser D., Shohat S., Gerda R., Nin Nun A., Shoshani A., Samra N.N., Verbeke W., Vrticka P., Ein-Dor T. (2026). The creative mind in the shadow of depressive symptoms: Epigenetic variation in HPA axis genes is associated with the link between depressive symptoms and creative personality. Psychoneuroendocrinology.

[18] Ising M., Maccarrone G., Brückl T., Scheuer S., Hennings J., Holsboer F., Turck C.W., Uhr M., Lucae S. (2019). FKBP5 gene expression predicts antidepressant treatment outcome in depression. Int. J. Mol. Sci..

[19] Beck A.T., Ward C.H., Mendelson M., Mock J., Erbaugh J. (1961). An inventory for measuring depression. Arch. Gen. Psychiatry.

[20] Kroenke K., Spitzer R.L., Williams J.B. (2001). The PHQ-9: Validity of a brief depression severity measure. J. Gen. Intern. Med..

[21] Hamilton M. (1960). A rating scale for depression. J. Neurol. Neurosurg. Psychiatry.

[22] Tung V.S., Thong N.V., Mai N.P., Linh L.T., Son D.C., Ha T.T., Hoa N.T., Long N.T., Tuan N.V. (2023). Screening severe depression of the Hamilton Depression Rating Scale, Hamilton Anxiety Rating Scale, Beck Depression Inventory Scale, and Zung’s Self-Rating Anxiety Scale among patients with recurrent depression disorder. Acta Inform. Med..

[23] Manea L., Gilbody S., McMillan D. (2012). Optimal cut-off score for diagnosing depression with the Patient Health Questionnaire (PHQ-9): A meta-analysis. CMAJ.

[24] Freedland K.E., Rich M.W., Skala J.A., Carney R.M., Dávila-Román V.G., Jaffe A.S. (2003). Prevalence of depression in hospitalized patients with congestive heart failure. Psychosom. Med..

[25] Kroenke K., Spitzer R.L., Williams J.B., Löwe B. (2010). The Patient Health Questionnaire somatic, anxiety, and depressive symptom scales: A systematic review. Gen. Hosp. Psychiatry.

[26] Jha M.K., Qamar A., Vaduganathan M., Charney D.S., Murrough J.W. (2019). Screening and management of depression in patients with cardiovascular disease: JACC State-of-the-Art Review. J. Am. Coll. Cardiol..

[27] Sulić P., Ražić Pavičić A., Đapić Ivančić B., Božina T., Božina N., Živković M. (2025). Role of genetic and epigenetic biomarkers in treatment-resistant depression: A literature review. Genes.

[28] Figueiredo S., Devezas M. (2024). Bridging the Gaps in Understanding POD and POCD: A thorough examination of genetic and clinical biomarkers. Perioper. Care Oper. Room Manag..

[29] Stępień K., Furczyńska P., Zalewska M., Nowak K., Włodarczyk A., Owsianka I., Skorek P., Zalewski J., Nessler J. (2021). Dementia screening in elderly high-risk patients following heart failure decompensation may predict unfavorable long-term clinical outcomes. Minerva Cardiol. Angiol..

[30] Kokoszka A., Cichoń E., Obrębski M., Kiejna A., Rajba B. (2020). Cut-off points for Polish-language versions of depression screening tools among patients with Type 2 diabetes. Prim. Care Diabetes.

[31] Kożybska M., Wojtkowska A., Szpakow A., Fedortsiv O. (2025). Predicting depressive symptoms among Polish university students through sociodemographic and lifestyle factors. Sci. Rep..

[32] Ślusarska B.J., Nowicki G., Piasecka H., Zarzycka D., Mazur A., Saran T., Bednarek A. (2019). Validation of the Polish language version of the Patient Health Questionnaire-9 in a population of adults aged 35–64. Ann. Agric. Environ. Med..

[33] Wiglusz M.S., Landowski J., Michalak L., Cubała W.J. (2016). Validation of the Polish version of the Hamilton Rating Scale for Depression in patients with epilepsy. Epilepsy Behav..

[34] Martiadis V., Raffone F., Atripaldi D., Testa S., Giunnelli P., Cerlino R., Russo M., Martini A., Pessina E., Cattaneo C.I. (2025). Gender differences in suicidal and self-harming responses to esketamine: A real-world retrospective study. Curr. Neuropharmacol..

[35] Schiffer A.A., Pelle A.J., Smith O.R., Widdershoven J.W., Hendriks E.H., Pedersen S.S. (2009). Somatic versus cognitive symptoms of depression as predictors of all-cause mortality and health status in chronic heart failure. J. Clin. Psychiatry.

[36] Smith O.R., Kupper N., Schiffer A.A., Denollet J. (2012). Somatic depression predicts mortality in chronic heart failure: Can this be explained by covarying symptoms of fatigue?. Psychosom. Med..

[37] Rollman B.L., Herbeck Belnap B., Mazumdar S., Houck P.R., He F., Alvarez R.J., Schulberg H.C., Reynolds C.F., McNamara D.M. (2012). A positive 2-item Patient Health Questionnaire depression screen among hospitalized heart failure patients is associated with elevated 12-month mortality. J. Card. Fail..

[38] Thombs B.D., de Jonge P., Coyne J.C., Whooley M.A., Frasure-Smith N., Mitchell A.J., Zuidersma M., Eze-Nliam C., Lima B.B., Smith C.G. (2008). Depression screening and patient outcomes in cardiovascular care: A systematic review. JAMA.

[39] Karege F., Perret G., Bondolfi G., Schwald M., Bertschy G., Aubry J.M. (2002). Decreased serum brain-derived neurotrophic factor levels in major depressed patients. Psychiatry Res..

[40] Monteleone P., Serritella C., Martiadis V., Maj M. (2008). Decreased levels of serum brain-derived neurotrophic factor in both depressed and euthymic patients with unipolar depression and in euthymic patients with bipolar I and II disorders. Bipolar. Disord..

[41] Yang X., Zhang M., Xie B., Peng Z., Manning J.R., Zimmerman R., Wang Q., Wei A.C., Khalifa M., Reynolds M. (2023). Myocardial brain-derived neurotrophic factor regulates cardiac bioenergetics through the transcription factor Yin Yang 1. Cardiovasc. Res..

[42] Nowak K., Stępień A., Furczyńska P., Zalewska M., Kanclerz G., Yika A.D.C., Stepien K., Nessler J., Zalewski J. (2022). Screening diagnosis of dementia in patients following heart failure decompensation is associated with a higher relative wall thickness. Bratisl. Lek. Listy.

[43] Sherwood A., Blumenthal J.A., Trivedi R., Johnson K.S., O’Connor C.M., Adams K.F., Dupree C.S., Waugh R.A., Bensimhon D.R., Gaulden L. (2007). Relationship of depression to death or hospitalization in patients with heart failure. Arch. Intern. Med..

[44] Moraska A.R., Chamberlain A.M., Shah N.D., Vickers K.S., Rummans T.A., Dunlay S.M., Spertus J.A., Weston S.A., McNallan S.M., Redfield M.M. (2013). Depression, healthcare utilization, and death in heart failure: A community study. Circ. Heart Fail..

[45] Celano C.M., Villegas A.C., Albanese A.M., Gaggin H.K., Huffman J.C. (2018). Depression and anxiety in heart failure: A Review. Harv. Rev. Psychiatry.

[46] Dowlati Y., Herrmann N., Swardfager W., Liu H., Sham L., Reim E.K., Lanctôt K.L. (2010). A meta-analysis of cytokines in major depression. Biol. Psychiatry.

[47] Barlattani T., Venturiello D., Grassi D., Romano S., Pacitti F. (2026). Bridging the heart-mind divide: Psychiatric implementation of the 2025 ESC mental-CVD consensus. Eur. Psychiatry.

[48] Monteleone P., Fabrazzo M., Martiadis V., Serritella C., Pannuto M., Maj M. (2005). Circulating brain-derived neurotrophic factor is decreased in women with anorexia and bulimia nervosa but not in women with binge-eating disorder: Relationships to co-morbid depression, psychopathology and hormonal variables. Psychol. Med..

[49] Duc S., Rainfray M., Soubeyran P., Fonck M., Blanc J.F., Ceccaldi J., Cany L., Brouste V., Mathoulin-Pélissier S. (2017). Predictive factors of depressive symptoms of elderly patients with cancer receiving first-line chemotherapy. Psychooncology.

[50] O’Connor C.M., Jiang W., Kuchibhatla M., Silva S.G., Cuffe M.S., Callwood D.D., Zakhary B., Stough W.G., Arias R.M., Rivelli S.K. (2010). Safety and efficacy of sertraline for depression in patients with heart failure: Results of the SADHART-CHF (Sertraline Against Depression and Heart Disease in Chronic Heart Failure) trial. J. Am. Coll. Cardiol..

[51] Angermann C.E., Gelbrich G., Störk S., Gunold H., Edelmann F., Wachter R., Schunkert H., Graf T., Kindermann I., Haass M. (2016). MOOD-HF Study Investigators and Committee Members. Effect of escitalopram on all-cause mortality and hospitalization in patients with heart failure and depression: The MOOD-HF randomized clinical trial. JAMA.

[52] Blumenthal J.A., Babyak M.A., O’Connor C., Keteyian S., Landzberg J., Howlett J., Kraus W., Gottlieb S., Blackburn G., Swank A. (2012). Effects of exercise training on depressive symptoms in patients with chronic heart failure: The HF-ACTION randomized trial. JAMA.

[53] da Cunha L.L., Feter N., Alt R., Rombaldi A.J. (2023). Effects of exercise training on inflammatory, neurotrophic and immunological markers and neurotransmitters in people with depression: A systematic review and meta-analysis. J. Affect. Disord..

