# Supplementary material for: Decreased Brain-Derived Neurotrophic Factor, Increased FK506 Binding Protein 5 and Female Sex Are Associated with Multiscale-Assessed Depressive Symptoms During Heart Failure Decompensation: A Polish Prospective Cohort Study
Source: J Clin Med. 2026 Oct 13;15(20):7893. doi: 10.3390/jcm15207893 (PMC13705762; doi:10.3390/jcm15207893)
Supplement: Supplementary file 1 [file jcm-15-07893-s001.zip › jcm-4505600-supplementary.pdf]

Supplementary Table S1. Characteristics of patients with (>11 points) and without (≤11 points) a positive PHQ-9 screening.

	**PHQ-9-defined SDD (n=13)**	**PHQ-9-defined No SDD (n=38)**	**P-value**
Clinical characteristics			
Age, years	70.0 (56.0–76.0)	69.0 (61.0–73.8)	0.829
Female sex	5 (38.5)	6 (15.8)	0.121
Body mass index, kg/m²	25.6 (23.0–29.1)	28.5 (26.0–30.2)	0.222
NYHA III/IV	11 (91.7)	29 (87.9)	0.706
Ischemic etiology	8 (61.5)	19 (50.0)	0.691
Prior myocardial infarction	7 (53.8)	15 (39.5)	0.563
Prior stroke	0 (0)	3 (7.9)	0.561
Arterial hypertension	11 (84.6)	33 (86.8)	1.000
Dyslipidemia	9 (69.2)	36 (94.7)	**0.031**
Diabetes mellitus	5 (38.5)	21 (55.3)	0.469
Renal failure	3 (23.1)	11 (28.9)	1.000
Peripheral arterial disease	2 (15.4)	9 (23.7)	0.706
Laboratory			
NT-proBNP, pg/mL	7436 (2386–14103)	4773 (2108–9187)	0.381
Hemoglobin, g/dL	13.6 ± 2.1	13.6 ± 1.8	0.993
Creatinine, µmol/L	97.0 (74.0–129.0)	101.0 (81.0–135.0)	0.808
C-reactive protein, mg/L	20.4 (7.2–33.1)	7.0 (2.7–11.7)	0.077
Total cholesterol, mmol/L	4.2 ± 1.3	4.0 ± 1.2	0.564
LDL cholesterol, mmol/L	2.3 ± 1.1	2.4 ± 0.9	0.697
Triglycerides, mmol/L	1.3 (1.1–2.2)	1.1 (0.9–1.4)	0.141
Albumin, g/L	37.5 ± 5.0	37.9 ± 4.6	0.804
Total protein, g/L	64.9 ± 9.2	64.2 ± 8.5	0.841
IL-6, pg/mL	150.8 (63.8–225.6)	78.6 (0–144.3)	**0.044**
BDNF, ng/mL	4.48 ± 1.37	4.73 ± 1.20	0.571

FKBP5, ng/mL	0.5 (0.2–1.0)	0.2 (0.1–0.6)	0.226
Echocardiography			
LV ejection fraction, %	30.0 (25.0–35.0)	25.0 (20.0–34.5)	0.291
LV end-diastolic diameter, mm	60.2 ± 6.7	62.1 ± 7.9	0.389
Interventricular septum, mm	10.0 (9.0–11.0)	11.0 (9.0–12.0)	0.181
LV mass index, g/m²	121.5 ± 23.5	133.0 ± 39.7	0.218
LV end-diastolic volume, mL	190.7 ± 51.6	207.5 ± 42.9	0.481
Global longitudinal strain, %	-8.0 ± 3.5	-7.7 ± 3.6	0.841
Left atrium diameter, mm	45.7 ± 5.4	46.6 ± 7.2	0.623
TAPSE, mm	18.6 ± 3.9	18.7 ± 5.7	0.964
RVSP, mmHg	38.0 (28.0–48.0)	34.0 (18.0–45.8)	0.430

Abbreviations: data are shown as mean ± SD, median (interquartile range) or number (percentage). NYHA: New York Heart Association, SDD: screening diagnosis of depression based on the PHQ-9 with more than 11 points, NT-proBNP: N-terminal prohormone of brain natriuretic peptide, LDL: low-density lipoprotein cholesterol, IL-6: interleukin 6, BDNF: brain-derived neurotrophic factor, FKBP5: FK506 binding protein 5, LV: left ventricular, TAPSE: tricuspid annular plane systolic excursion, RVSP: right ventricular systolic pressure.
